# Locoregional Flap Reconstruction Following Oromaxillofacial Oncologic Surgery in Dogs and Cats: A Review and Decisional Algorithm

**DOI:** 10.3389/fvets.2021.685036

**Published:** 2021-05-21

**Authors:** Michel Guzu, Diego Rossetti, Philippe R. Hennet

**Affiliations:** ^1^Dentistry and Oromaxillofacial Surgery Unit, Department of Surgery, ADVETIA Centre Hospitalier Vétérinaire, Vélizy-Villacoublay, France; ^2^Department of Surgery, CHV ADVETIA, Vélizy-Villacoublay, France

**Keywords:** oral, maxillofacial, flap, surgery, reconstruction, oncology, dogs, cats

## Abstract

Primary treatment of most oromaxillofacial tumors in dogs and cats is resective surgery. Management of malignant tumors may be very challenging as wide/radical free-margin surgical removal must be achieved while preserving vital functions. Removal of orofacial tumors may result in large defects exposing the oral cavity or creating a communication with the nasal, pharyngeal, or orbital cavities. Such defects require orofacial reconstruction in order to restore respiratory and manducatory functions. The veterinary surgeon must be familiar with reconstructive techniques in order to prevent the inability of closing the defect, which could lead to an insufficient resection. Small oral defects exposing the nasal cavity are best closed with local random mucosal flaps. Closure of large oral defects may be better achieved with a facial or major palatine-based axial-pattern flap. Small to moderate facial defects can be closed with local advancement or transposition skin flaps. Reconstruction of large facial defects often requires the use of locoregional axial pattern flaps such as the caudal auricular, the superficial temporal, or the facial (angularis oris) myocutaneous axial pattern flaps. Recent publications have shown that the facial (angularis oris) flap is a very versatile and reliable flap in orofacial reconstructive surgery. A surgical decision algorithm based on the size, nature, and location of the defect is proposed.

## Introduction

Oral tumors are common in dogs and cats, representing 1.2–10% of all tumors (1, 2). The incidence of oral tumours has been calculated to be 4.9 per 1,000 dogs (0.5%) and 4.9 per 1,000 cats (0.5%) and malignant tumours represented 53.6% of the canine oral tumours and 58.1% of the feline oral tumours identified in a recent demographic study (3). Malignant melanoma, squamous cell carcinoma, and fibrosarcoma in dogs, and squamous cell carcinoma in cats are the most common oral malignancies (4, 5).

Though a lot of progress has been made in adjunct therapy, surgery is still the primary treatment for most orofacial tumours in humans as well as in dogs and cats (6–12). Modern diagnostic imaging technology such as CT scan, MRI and, more recently, ^18^FDG-PET CT has contributed to better evaluation of local, regional, and distant extent of disease and better surgical and radiation appraisal (13–29). Nevertheless, in our experience, oral tumours in dogs and cats are often first discovered, or referred, late. Studies in humans have shown that the greater diagnostic delay for oral tumours, the more advanced the disease is at staging. Advanced disease requires more radical surgery and results in a poorer outcome (9, 30, 31). Despite the progress made in adjunct therapy (radiation therapy, chemotherapy, immunotherapy, targeted therapy), wide/radical resection of bone and soft tissue is often required (9, 11). Curative-intent maxillofacial ablative (oncologic) surgery is aimed at achieving an en-bloc free-margin resection (R0 resection). Insufficient resection, either microscopically positive margins (R1 resection) or macroscopically cutting through the tumour (R2 resection), constitutes high-risk situations leading to relapse and potential neoplastic spread (6, 7, 11, 12, 32–38). While achieving clean margins (R0 resection) is mandatory, avoiding the unnecessary sacrifice of normal tissue and preserving respiratory and oral functions are also of fundamental importance (37, 39). Cosmesis (preservation or restoration of form) seems less of a concern in dogs and cats compared to that in humans, though in our experience acceptance of the surgery by owners may be strongly influenced by the post-operative appearance. The major risk for the surgeon is the fear of being unable to close the surgical defect in a functional and/or cosmetic manner. Having this concern in mind while performing the surgery will negatively affect the extent of the ablation and increase the risk of relapse. As the veterinary surgeon is most of the time both the maxillofacial surgeon performing the resective surgery and the plastic surgeon performing the reconstructive surgery, knowledge of surgical techniques enabling the closure of large hard and soft tissue defects is mandatory in order to achieve the best results. Ideally, the options for reconstruction are thoroughly discussed and decided prior to performing the ablative surgery. Improvement in median survival and progression-free survival in recent publications may be due to earlier detection of the disease, better evaluation of its extent but also to a more aggressive surgery and adjunct treatments (11).

Orofacial reconstructive techniques used in veterinary medicine include local and regional random flaps, tension relieving techniques, local and regional pedicle flaps, free partial/total skin grafts, and vascularized free flaps (40–44). Various decisional algorithms have been published over time in human head and neck reconstructive surgery, with main criteria based on the location and the size of the defect. However, no consensus has been made or generally accepted in the veterinary field to date (45–51). Vascularized free flaps, either simple or composite, constitute the gold standard treatment for the reconstruction of most large defects in humans, with good clinical outcome achieved in up to 98% of the cases. Specific contraindications may include some medical conditions (e.g., atherosclerosis, on-going tobacco consumption) or elderly patients (52, 53). Despite recipient vessels of the head and neck having been studied in dogs, only very few clinical cases of orofacial reconstruction with a free flap have been reported in the veterinary literature (54–57). Slow development of these techniques in the veterinary field may be due to the prolonged time and cost inherent to such surgeries, the need for highly trained surgeons with microsurgical skills, specific instrumentation and monitoring, and appropriate facilities equipped with intensive care units (44, 58, 59). Nevertheless, recent trends in orofacial plastic and reconstructive surgery are reconsidering locoregional flaps in terms of functional outcomes, complication rate, cost, and prognosis in some clinical situations in humans (53, 60).

## Cutaneous and Mucosal Vascularization of the Oromaxillofacial Region

**Figure 1:**
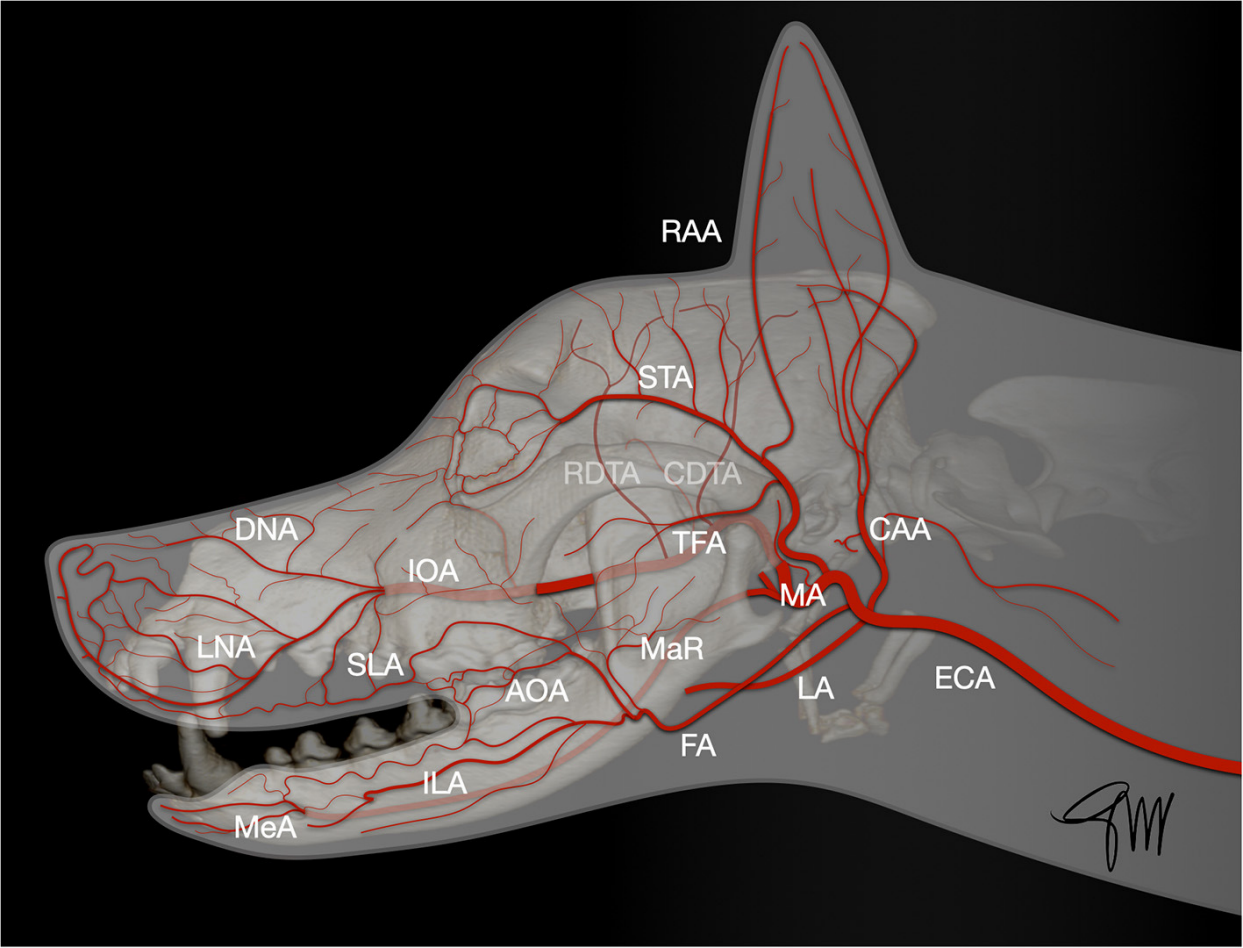
Main arterial blood supply to the head in the dog. AOA, angularis oris artery; CAA, caudal auricular artery; CDTA, caudal deep temporal artery; DNA, rostral dorsal nasal artery; ECA, external carotid artery; FA, facial artery; ILA, inferior labial artery; IOA, infraorbital artery; LA, lingual artery; LNA, lateral nasal artery; MA, maxillary artery; MaR, masseteric ramus; MeA, mental artery; RAA, rostral auricular artery; RDTA, rostral deep temporal artery; LA, superior labial artery; STA, superficial temporal artery; TFA, transverse facial artery.

The skin (cutis) consists of a superficial epidermis of stratified squamous epithelium and an underlying connective tissue, the dermis (corium) separated by the basement membrane. The dermis contains blood vessels, lymphatics, muscles, and nerve endings. A subcutis or hypodermis, composed of fat and loose collagenous trabecular and elastic fibers, connects the dermis with the fascia and acts as a moveable support allowing the skin to glide over the underlying tissue. Cutaneous or panniculus muscles are attached to the dermis and are anchored to the subcutaneous fascia rather than to the bone. In the head and neck area, they comprise the superficial craniofacial muscles, the platysma, the sphincter colli superficialis, and the sphincter colli profundus (61, 62). Main regional arteries give off perforator branches traversing the skeletal muscles to supply the subdermal plexus. The arteries to the skin include simple direct cutaneous arteries running between muscles toward the skin and musculocutaneous (mixed cutaneous) arteries supplying both muscles and skin (63–65). In mucocutaneous regions, such as the lip and the cheek, there is a transition from the integument to the mucous membrane, which is a stratified epithelium lining the oral cavity. The cutaneous portion is typically supplied by three arterial and venous plexuses: (1). the deep or subcutaneous plexus providing the adipose and areolar tissue on the deep face of the dermis as well as the superficial muscles of the head, (2). the middle plexus layer located below or at the level of the sebaceous glands, (3). The superficial plexus layer lying in the outer layers of the dermis. In the mucous membrane, the vessels form a superficial plexus supplied by a meshwork in which there may be layers suggestive of a middle and deep plexuses; large vessels lie on the oral surface of the orbicularis oris muscle (65). Arteries supplying the orofacial region originate from the external carotid artery. Main branches are the ascending pharyngeal, lingual, facial, caudal auricular, superficial temporal, and maxillary arteries and its terminal branches (66) (Figure 1).

### Upper Face and Neck

The ***caudal auricular artery*** arises from the base of the annular cartilage. Among its branches, the sternocleidomastoideus supplies the cleidocephalic and sternocephalic muscles and also supplies the cranial part of the cervical skin, the platysma and subcutaneous fat (66, 67). This cutaneous branch originates in the area between the lateral aspect of the wing of the atlas and the vertical ear canal. Then it runs ventrocaudally toward the scapula, and eventually anastomoses with some cutaneous branches originating from the omocervical artery (67).

The ***superficial temporal artery*** provides blood supply to the skin and frontalis muscle in the temporal region in dogs and cats. It arises rostral to the base of the auricular cartilage and extends dorsally toward the zygomatic arch. From the base of the zygomatic arch, it extends rostrally along the zygomatic arch toward the upper eyelid (66, 68). A direct cutaneous branch of the superficial temporal artery is observed where the artery and its accompanying vein enter the temporal fascia (69).

### Middle and Lower Face

The main arteries supplying the middle and lower face, are the facial artery and the infraorbital artery (70). They originate from the external carotid artery (71). Veins follow a similar path, nearby the arterial web, eventually draining in the maxillary and linguofacial veins, themselves merging in the external jugular vein, that constitutes the main collecting vessel of the head (65, 66, 70–72). After sending off the inferior labial artery, just rostral to the masseter muscle and medial to the digastric muscle, at the level of the of the mandibular lymph nodes, the facial artery runs caudo-rostrally between the platysma and the buccinator muscle into the cheek opposite to the last molar teeth and divides into the angularis oris and superior labial arteries; the angularis oris is usually thicker at its origin than the superior labial artery (66, 70, 71). The ***angularis oris artery*** runs rostrally and divides at the labial commissure into a superficial and a deep ramus. It supplies in part the buccinator and orbicularis oris muscles as well as the skin and mucosa of the commissure and caudal part of the lips (66). The superficial (cutaneous) ramus is thinner than the deep one and divides into a superior and inferior branch, which anastomoses with the superior and inferior labial arteries, supplying the skin and the orbicularis oris of the caudal part of the superior and inferior lips. The deep (mucosal) ramus of the angularis oris has a course similar to the superficial ramus and has a broader distributing territory on the oral mucosa than on the skin. It also anastomoses with the superior labial artery, forming an arterial arch, and to the inferior labial artery (70). The ***superior labial artery*** is the termination of the facial artery. It runs rostrally in the superior lip and anastomoses at the level of the second and third maxillary premolar teeth with the superior ramus of the infraorbital artery forming an arterial arch (70). It also sends branches caudally to the eyelid and to the dorso-rostral part of the masseter, one of these branches anastomosing with the transverse facial artery, and ventrally to the superior branches of the angularis oris (70). The caudal half of the superior lip is vascularized by the superior labial artery. This vascular territory is connected through anastomoses to the rostral half supplied by the infraorbital artery (72). The ***inferior labial artery*** runs rostrally along the orbicularis oris muscle from the ventral border of the mandible just rostral to the masseter muscle. It supplies ~60% of the lower lip (72). In the rostral part of the lower lip, the inferior labial artery anastomoses with the caudal mental branch of the inferior alveolar artery at the caudal mental foramen (66). The ***infraorbital artery*** supplies the rostral half of the superior (maxillary) lip. It leaves the maxillary bone through the infraorbital foramen. In the infraorbital canal or near the foramen it branches into the dorsal nasal artery. About 1–2 cm rostral to the foramen, the artery changes direction and enters the superior lip passing in the mucosa at the level of the first maxillary premolar tooth. At that level, it releases one or two lateral nasal arteries and continues as the dorsal anterior nasal artery. Numerous anastomoses occur between these branches forming a dense arterial network in the rostral part of the nose (70).

## Principles of Flaps

A flap (pedicle graft) is a transfer of tissue that has its own blood supply. In contrast with a non-vascularized graft, a flap is less dependent on the recipient site supply as it brings its own. It allows more tissue bulk and predictable healing and viability. Based on the main pattern of their blood supply, two types of flaps are recognized: random and axial pattern flaps (64, 73).

### Random Pattern Flap

A random pattern flap is a local flap relying on general subdermal (submucosal) blood vessels. Because there is no specific artery supplying the flap, risk of necrosis at the distal end of the flap is increased and narrow flaps need to be avoided. To prevent this, the general guideline is a ratio 2:1 for base to length of the flap. Owing to the very good vascularisation of orofacial structures, random pattern flaps on the face can sustain a 3:1 length to width ratio (74). To compensate for excessive shrinkage after harvest, the soft tissue flap should be designated to be 10–20% larger than the defect to cover (75). Because they only rely on the subdermal plexus for survival, they should not be fenestrated, tacked down with walking sutures, or stretched excessively under tension. Excess of tissue (dog's ear) at the base of the flap should not be removed during initial closure of the wound (41).

The type of primary movement or motion of the flap can further subdivide random pattern flaps into three categories: advancement, rotation, and transposition. Advancement and rotation flaps, also called sliding flaps, recruit adjacent lax tissue and move in, respectively, either a linear or arced motion to fill in the primary defect. Transposition flaps, also called lifting flaps, recruit non-contiguous donor tissue incised, and lifted over intact tissue and placed into the primary defect (76, 77).

In a recent retrospective study, the complication rate for random flaps in the face and head area was 17%, with no major complications (>50% flap failure or requiring a second surgery) reported. This was significantly less than for skin deficits of the torso or limbs (78). The most common complication was dehiscence of the distal wound edge. Advancement and transposition flaps had lower complication rates than rotation flaps. The mean time (±SD) to onset of clinical signs of complications was 6.9 days (±4.0) (78).

### Axial Pattern Flap

The axial pattern flap is a pedicle graft that incorporates a direct cutaneous artery and vein into its base. The vessels extend up the length of the flap to a variable degree, the terminal branches of which supply blood to the subdermal/submucosal plexus. All the skin or mucosa supplied by this vascular territory, or angiosome, can be lifted from the donor site and will survive provided these vessels remain intact and patent (79). As a result of this, axial pattern flaps have better perfusion (80). Nevertheless, in a recent retrospective study reporting the outcome and complications of axial pattern skin flaps in dogs and cats, postoperative complications occurred in 83% of the cats and 92% of the dogs with 38% of the cats and 34% of the dogs requiring one or more additional surgeries. Wound dehiscence was the most common complication with 58 % of dogs and cats affected (81). Though the percentage of complications was high, most of them were minor complications. The outcome of the flap surgery was considered good to excellent in 58% of the cats and 66% of the dogs. Overall, 93% of the wounds were successfully reconstructed. As only a few surgeries involved facial/head flaps, unfortunately no specific conclusion can be drawn for maxillofacial reconstructive surgery (81).

Flaps may also be described in terms of proximity to the recipient site (local, regional, distant). Local flaps involve transfer of tissue from the immediate adjacent anatomic subsite. Regional flaps use tissue from a neighbouring site. Flaps where the bulk of tissue is limited to the head and neck region are classified as loco-regional flaps; they may be random or axial pattern flaps and may involve only one tissue component (e.g., oral mucosa, skin) or several tissue components such as mucosa, muscle, skin or bone harvested from the same angiosome (composite flaps).

## Reconstruction Techniques

### Oral Defects

Oral defects may involve bone and oral mucosa. Most of the time, bone reconstruction is not performed and coverage of the defect and restoration of the oral cavity lining is achieved through the use of mucosal or myomucosal flaps harvested from the alveolar and/or the labiobuccal mucosa or through the use of palatal mucoperiosteal flaps. These local flaps are supplied by branches of the facial artery (angularis oris and superior labial arteries) in the caudal (cheek) area, by the infraorbital artery and its ramus (lateral nasal) for the superior lip, by the inferior labial artery and mental artery for the inferior lip or by the major palatine for hard palate mucoperiosteal flaps. The local flaps may be random or axial pattern flaps depending whether direct artery and vein have been included in the base of the flap.

Large defects of the oral cavity may be mainly closed through local advancement, transposition flap or combination of, and sutured in a single or double-layer technique. Double-layer closure is considered to have a positive influence on the outcome of palatal defect repair and is advocated whenever possible (82). Palatal defects create a communication between the oral and nasal cavities. Therefore, single layer flap reconstruction of palatal defects is characterized by most of the inner surface of the flap facing the nasal passages without any underlying connective tissue support. Moreover, wound edges and suture lines may end up being located over the defect. To avoid this situation, reconstruction with two flaps overlying on each other, their connective tissue in contact, creates a water-tight seal. Creating a double layer of viable connective tissue offers better resistance to adverse oral conditions and provides better healing conditions by increasing support to suture lines, favouring revascularisation when the connective tissue of both flaps is in contact compared to when a single-layer flap is closed over the defect (82). In oral oncologic surgery, a two-flap double-layered technique may not be practical because of insufficient remaining soft tissue to close the defect. However, when using a single oral flap including submucosa or a myomucosal flap, a double-layered closure can be achieved by suturing the flap in two layers, a deep submucosal layer, and a superficial mucosal layer. Large flaps must be harvested while preserving the local (palatine, infraorbital, facial) vascularisation as much as possible. Blood vessels at the rostral end of the flap, which need to be transected for tension-free closure, are ligated with 3-0 or 4-0 polyglactin 910. Flaps are closed in a simple-interrupted pattern using a 5-0, 4-0, or 3-0 absorbable monofilament suture materials such as poliglecaprone 25.

**Figure 2:**
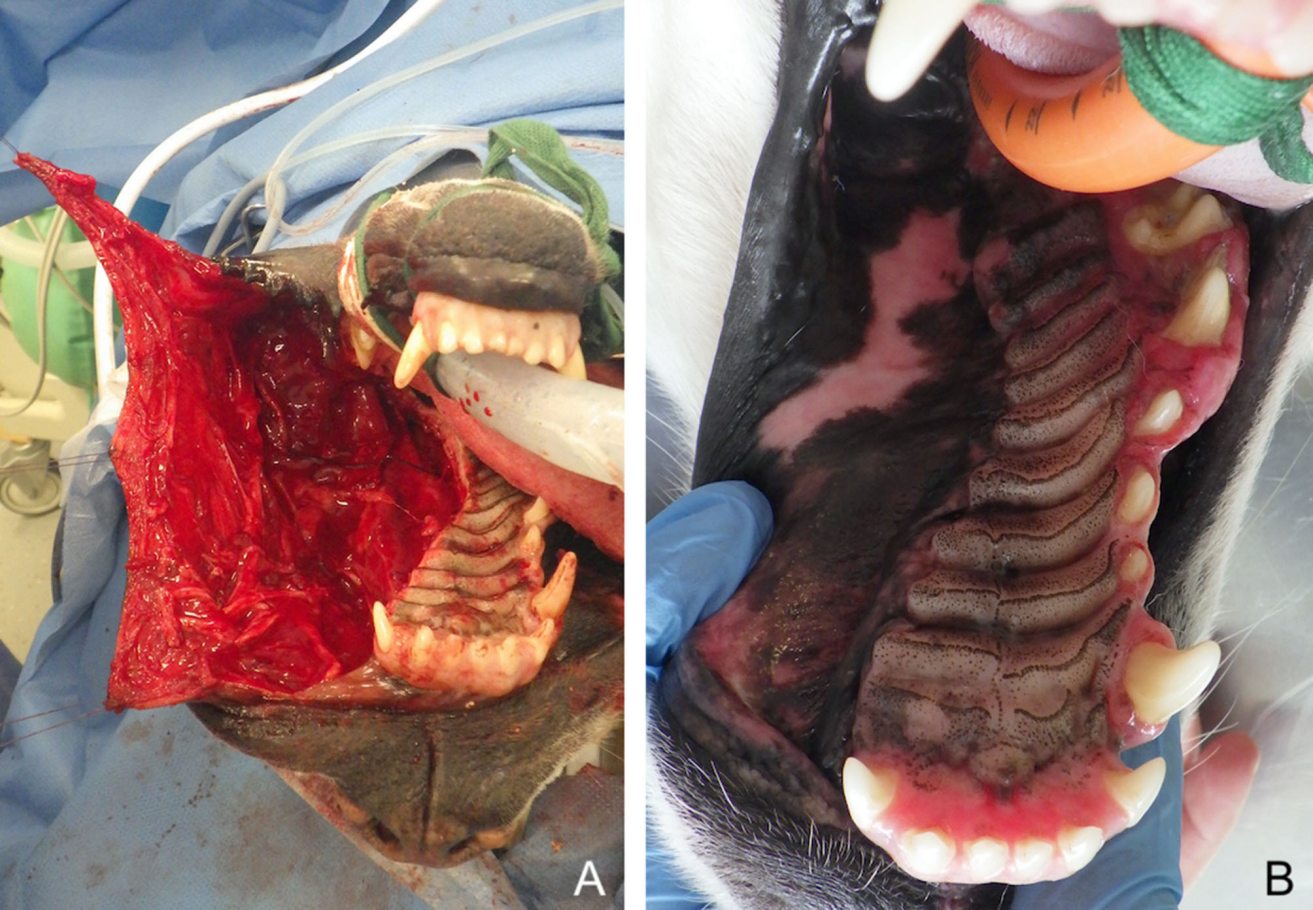
Large myomucosal advancement flap for closure of a unilateral maxillectomy defect (fibrosarcoma) in a dog. **(A)** Harvesting of the flap and manipulation with stay sutures. **(B)** 12-months postoperative aspect.

**Figure 3:**
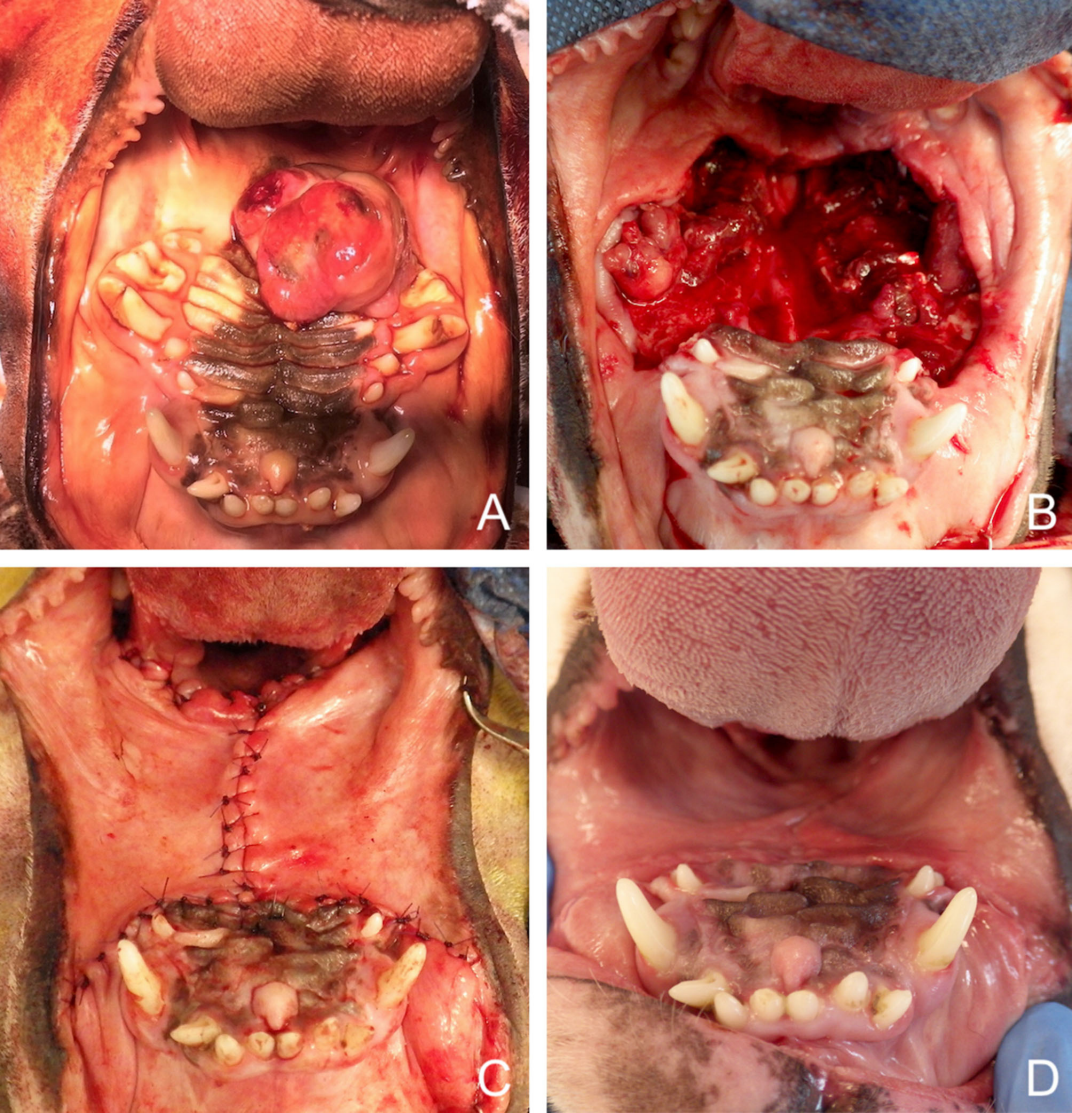
Bilateral myomucosal advancement flaps for closure of a large central maxillary defect after removal of a multilobular tumour in a dog. **(A)** Preoperative aspect of the tumour. **(B)** Radical maxillectomy. **(C)** Immediate postoperative aspect. **(D)** 5-months postoperative aspect.

***Advancement flaps*** can be used to repair a palatal defect by advancing the oral mucosa medially. The distance from the edge of the defect to the mucocutaneous junction is measured to evaluate whether sufficient amount of mucosa is available to cover the defect. Uni- or bilateral flaps may be used depending on the size and location of the defect. These full thickness mucosal advancement flaps are usually considered random flaps. Nevertheless, by preserving the main arterial supply (angularis oris, superior labial, lateral nasal, and their anastomoses) during careful deep submucosal dissection they may function similar to an axial pattern flap. Slightly divergent mucosal releasing incisions are made rostrally and caudally with a number 15 blade to design a slightly trapezoidal flap. In the caudal area, care is taken to preserve the angularis oris and superior labial vascularisation. In the rostral part, the lateral and/or dorsal nasal artery may be ligated and cut to enable medial displacement of the flap and tension-free closure. Flap elevation is performed with a periosteal elevator followed by blunt deep submucosal dissection with small curved scissors to the level of the mucocutaneous junction (83). The flap is manipulated with stay sutures and positioned in its final location to assess tension (Figure 2). When mucoperiosteum of the hard palate is still available after the resective surgery, a bi-pedicle mucoperiosteal flap may be advanced medially to decrease tension on the opposite oral mucosa advancement flap. Oral advancement flaps may be harvested from the entire lip and cheek and are, subsequently, adequate for closure of large defects (Figure 3). Their limitation is the width of oral mucosal tissue available, which may not be sufficient to fully cover a large central oral defect. Dogs with wide and loose lips harbour a greater width of oral mucosa, thus facilitating the surgical closure compared to dogs with a long nose and tight lips.

Several axial pattern transposition flaps have been described in the literature. In 1989, Bozola et al. first described an axial pattern buccinator myomucosal flap based on the buccal and facial arteries to treat oral defects in humans (84). In 1992, Pribaz et al. proposed a modification of Bozola's flap with the ***Facial Artery MyoMucosal (FAMM) Flap*** (85). The FAMM flap is considered a robust and versatile reconstructive option for small to medium size defects of the oral cavity or oropharynx in humans (53, 75, 86). Similarly, axial pattern myomucosal flaps harvested from the oral mucosa and based on collaterals of the facial artery, the angularis oris and/or superior labial arteries, respectively, have been described in dogs (72, 87). In their original description of the angularis oris axial pattern buccal flap in two dogs, Bryant et al. used a skin incision through the cheek to identify the vascular structure and design the buccal mucosal flap (87). Three other published cases reports, describing a total of five cases, used same surgical technique (88–90). A possible rostral advancement of the flap to the level of the canine tooth has been described when the flap is islandized (87). Nevertheless, islandization takes the risk of damage to the vascular supply of the flap at its base and cannot be systematically recommended (72). A wide based flap preserving the mucosal layer over the pedicle and avoiding the island design increases the chance of inclusion of venous drainage, which is fundamental for flap survival in the first weeks (76, 91–93). A longer myomucosal flap may be obtained by not only incorporating the angularis oris but also the superior labial artery. This superior labial myomucosal flap is designed as a peninsular flap with a caudal base in the retromolar area and can extend rostrally to the level of the canine tooth. It is harvested intraorally, after identification of the vascular supply through transillumination or the use of a handheld doppler, with no skin incision. The plane of dissection of the flap lies between the skin and the buccinator/orbicularis oris muscles. The flap also incorporates the superior labial vein. Based on a cadaveric vascular anatomic study, a good clinical outcome of this flap has been recently reported in one dog (72).

**Figure 4:**
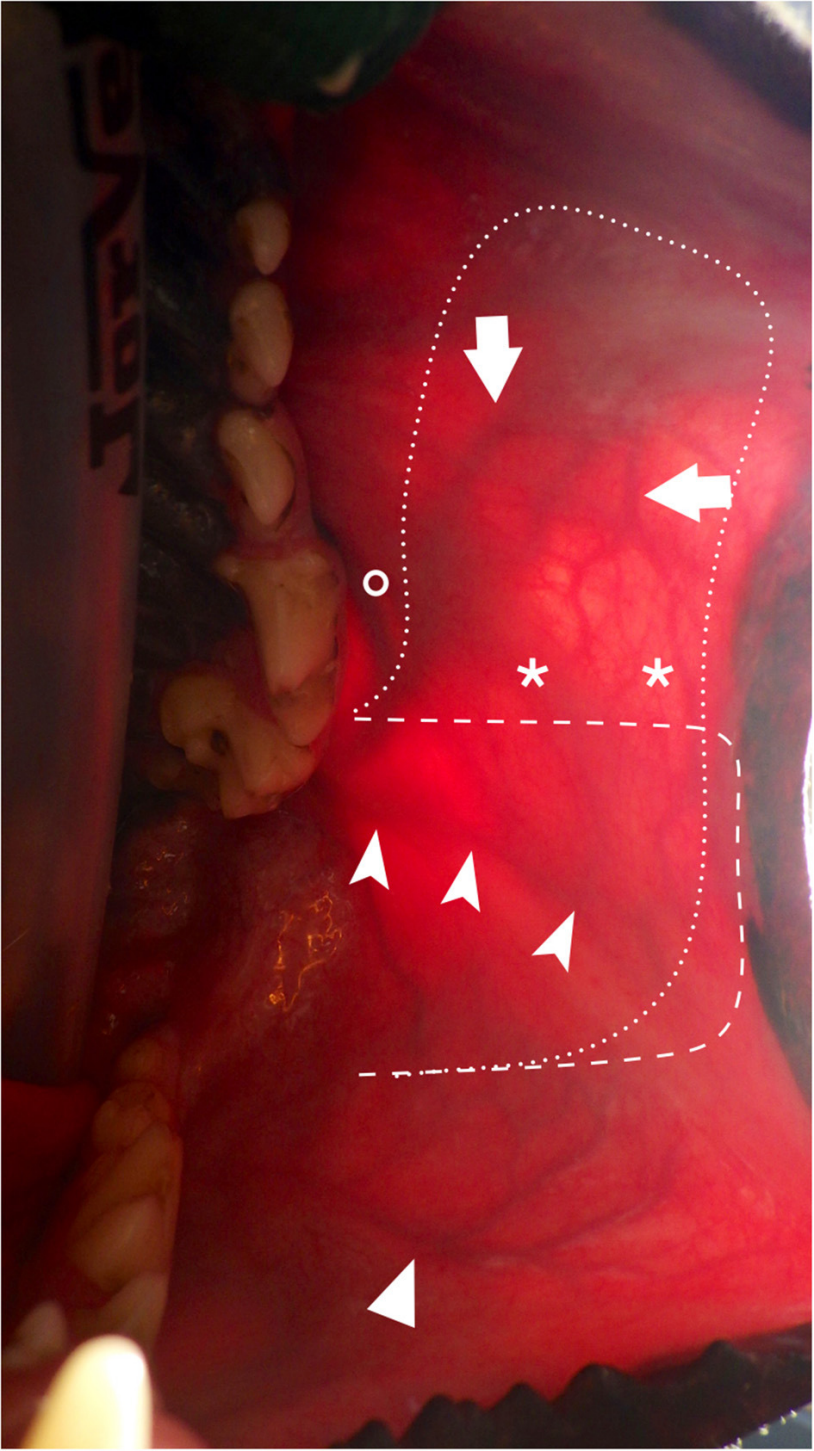
Transillumination intraoral view of the main arteries of the cheek and lips and surgical outline. (arrow: SLA; chevron arrowhead: AOA; simple arrowhead: ILA; asterisks: anatomoses between SLA and AOA; interrupted dash line: limits depicting the facial artery myomucosal (FAMM) flap centered on the AOA; interrupted dot line: limits depicting the facial artery myomucosal (FAMM) flap incorporating the AOA and SLA; circle: parotid duct papilla. AOA, angularis oris artery; SLA, superior labial artery; ILA: inferior labial artery.

**Figure 5:**
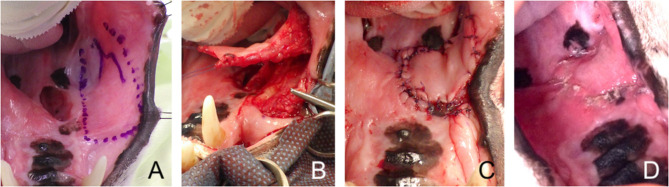
Facial artery myomucosal (FAMM) flap. **(A)** Revision surgery of a large palatal defect; surgical limits of the FAMM flap depicted with a surgical marker. **(B)** Harvesting of the myomucosal flap. **(C)** Immediate postoperative appearance. **(D)** 3-weeks postoperative aspect.

These two types of myomucosal flaps differ very little. They are peninsular flaps with a base located caudally, at the level of the mandibular ramus and the masseter muscle (retromolar area). Because they are both based on the facial artery and its collaterals, the board term of FAMM flap, similar to human terminology, could rather be used. The angularis oris artery is directed toward the commissure of the lip whether the superior labial artery continues beyond the commissure in a more dorsal direction along the superior lip and can subsequently be extended further rostrally (Figure 4). From the clinical point of view, incorporation of one or both vessels is based on the location and size of the defect to be covered. The commissure and the superior lip are retracted with stay sutures and the first step is to mark the flap on the oral mucosa with a surgical marking pen according to the predefined limits. Harvesting of the flap is made through incision with a n°15 scalpel blade of the mucosa, submucosa and buccinator/orbicularis oris muscles. The flap is elevated with dissection scissors in a layer underneath the superior labial and/or angularis oris artery, which is followed in a retrograde fashion toward the retromolar area. The artery is left attached to the overlying tissue over its entire length. To facilitate gentle dissection, stay sutures are placed on the distal edge of the flap. The flap can be transposed over the defect by rotating it up to a 90° angulation. Rotation up to 180° is theoretically possible, in case of oropharyngeal defects, but the arc of rotation should be minimal as during transposition over the palate its base needs to be twisted along its long axis (72). The main limitation of these myomucosal flaps is the width of cheek/lip mucosa available. Dogs with a long nose and narrow cheek and lips are less likely to provide a fair amount of mucosa compared to dogs with hanging lips. The width of mucosa located between the attached gingiva and the muco-cutaneous junction is the maximal width of the flap that can be used. The flap is either transposed in a parasagittal or transverse direction (90° rotation). When transposed along the long axis of the defect, the width of the flap should be larger than the width of the defect and the length of the flap larger than the length of the defect. When transposed with a 90° rotation, the width of the flap should be larger than the length of the defect and the length of the flap larger than the width of the defect. The donor site is either left exposed and undergoes secondary healing or is closed for primary healing. Closing of the donor site may result in a tight lip if most of the lip mucosa has been harvested. Whenever possible, at least 1 cm of oral mucosa is left at the mucocutaneous junction to facilitate closure of the donor site (Figure 5).

A modification of the ***superficial cervical myo-cutaneous flap*** (SCMC) has been studied by Dundas et al. for the closure of caudal partial-thickness defects located in the palate in an experimental setting (94). This flap is based on the prescapular perforating branch of the superficial cervical artery (formerly omocervical trunk), arising from the subclavian artery between the omotransversarius and trapezius muscles (66, 94). A 6–8-cm-wide islandized flap centered on this arterial branch and its corresponding vein and incorporating the contralateral angiosome was outlined. The vascular supply of the flap was studied on cadavers and a two-stage procedure was performed on 3 experimental dogs. In stage 1, the flap was elevated and its survival was observed after ligation of the opposite perforating blood vessel and de-epithelialization of its distal end. In stage 2, 7 days later, the necrotic tissue at the distal end of the flap was trimmed and the flap was implanted in the oral cavity through parapharyngeal tunnelization. The three flaps showed good survival post implantation with a small area of partial dehiscence at the rostral corner of the flap in one dog. Nevertheless, it should be noticed that in this experimental setting only partial defects of the caudal oral cavity were studied and that a loss of 20–25% of the originally anticipated flap length was observed presumably secondary to the two-staged protocol (94). As to our knowledge the outcome of this procedure in a clinical setting has not been further documented, caution is advised prior to extrapolating this protocol to clinical situations with large oronasal defects.

### Facial Defects

Oral tumours with aggressive behaviour may require lip/cheek resection to achieve tumour-free margins. Various flap techniques may be used to close the full-thickness defect and restore function. Ideally, both the mucosal and the skin layer need to be reconstructed. This is mostly possible with the use of local flaps providing skin and oral mucosa from adjacent lip and cheek. When using loco-regional flaps to close large lip/cheek defects, oral mucosa may not be available in sufficient quantity. Options for reconstruction of the oral mucosal lining include using the remaining nearby oral mucosa resulting in a tighter lip with a shorter vestibule, closing the defect with skin only without a mucosal layer, which may result in wound contraction and poorer cosmetic results, using a mucosal graft or using an inversed skin flap (41, 95).

Delicate technique and proper flap design are essentials to avoid tissue damage and ischemia. The flap must be handled with care favouring the use of stay sutures rather than toothed forceps or skin hooks to hold the margins (96, 97). A scalpel blade is preferred to monopolar electrosurgery for skin incisions as the latter has been shown in dogs to be associated with delayed healing and more complications within the first 7 days (98). Adequate undermining and sutures are other essential components of a successful tension-free closure. Sutures should not be too tight as with the development of post-operative edema they may strangulate tissue margin and induce ischemia (96).

Most *local flaps* are based over the subdermal plexus circulation unless a direct artery and vein (perforating vessels) are fortuitously included in the base of the flap (becoming an axial pattern flap) (95). Closing of the defect implies first realigning the mucocutaneous junction by placing a single interrupted absorbable suture on the mucosal side at the distal tip of the flap, closing the oral mucosa as a first layer with interrupted absorbable sutures and the skin as a second layer with interrupted non-absorbable sutures. In large dogs, a third intermediate suture layer can be placed on the superficial muscular layer. Removal of the skin sutures around the mouth, especially in cats or dogs with bad behaviour, may require sedation/anaesthesia. To avoid this trouble and for cost-effectiveness, the poliglecaprone-25 suture material remaining after closure of the mucosa can be used to close the skin layer using either an intradermal or a cutaneous suture pattern. No statistically significant cosmetic difference and no statistically significant difference in wound complications (infection, hematoma or dehiscence) have been shown in facial surgery in humans between skin closure with poliglecaprone-25 or polypropylene (99, 100).

**Figure 6:**
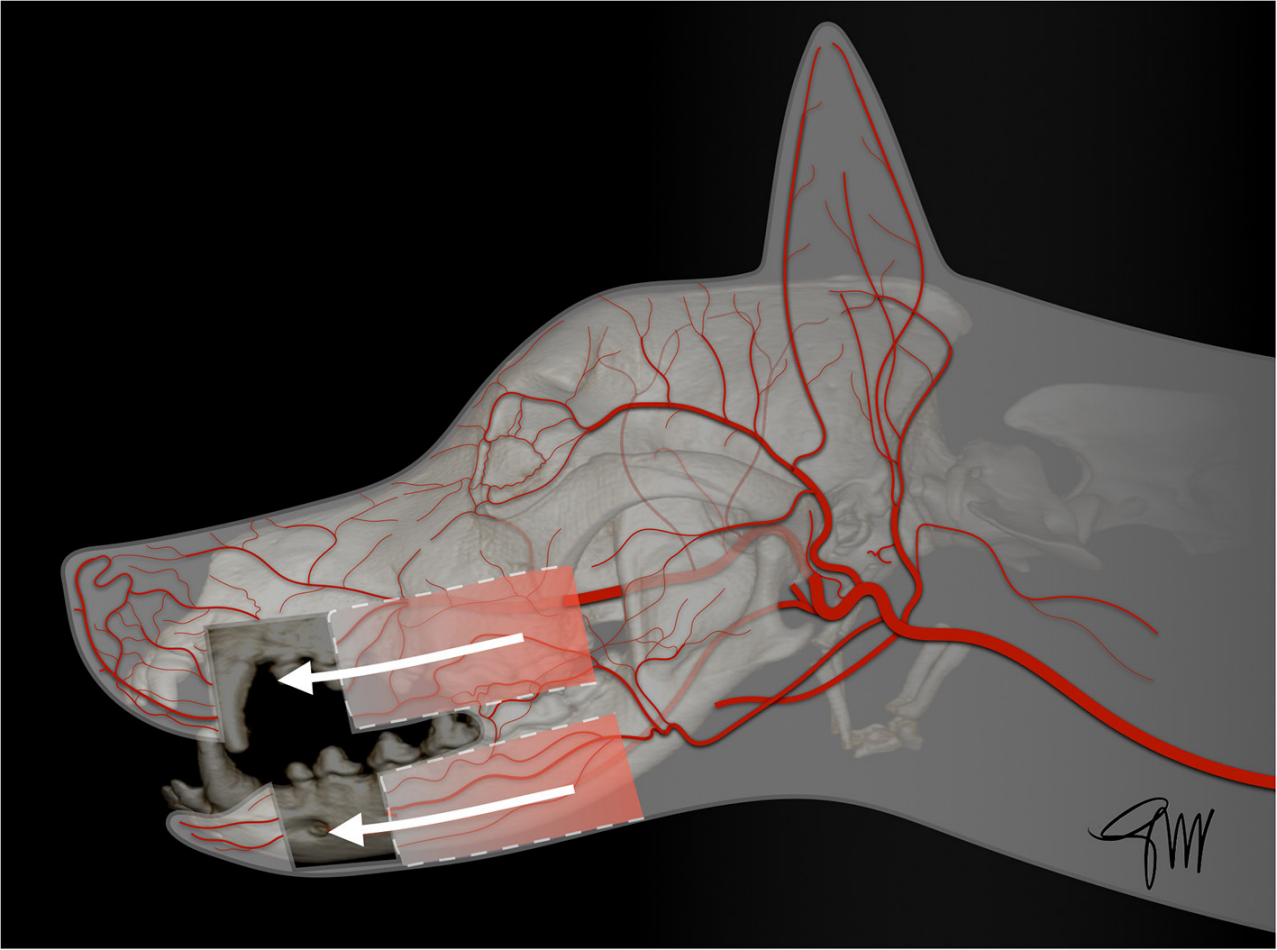
Drawing depicting full-thickness labial/buccal advancement flaps for upper or lower lip defects.

**Figure 7:**
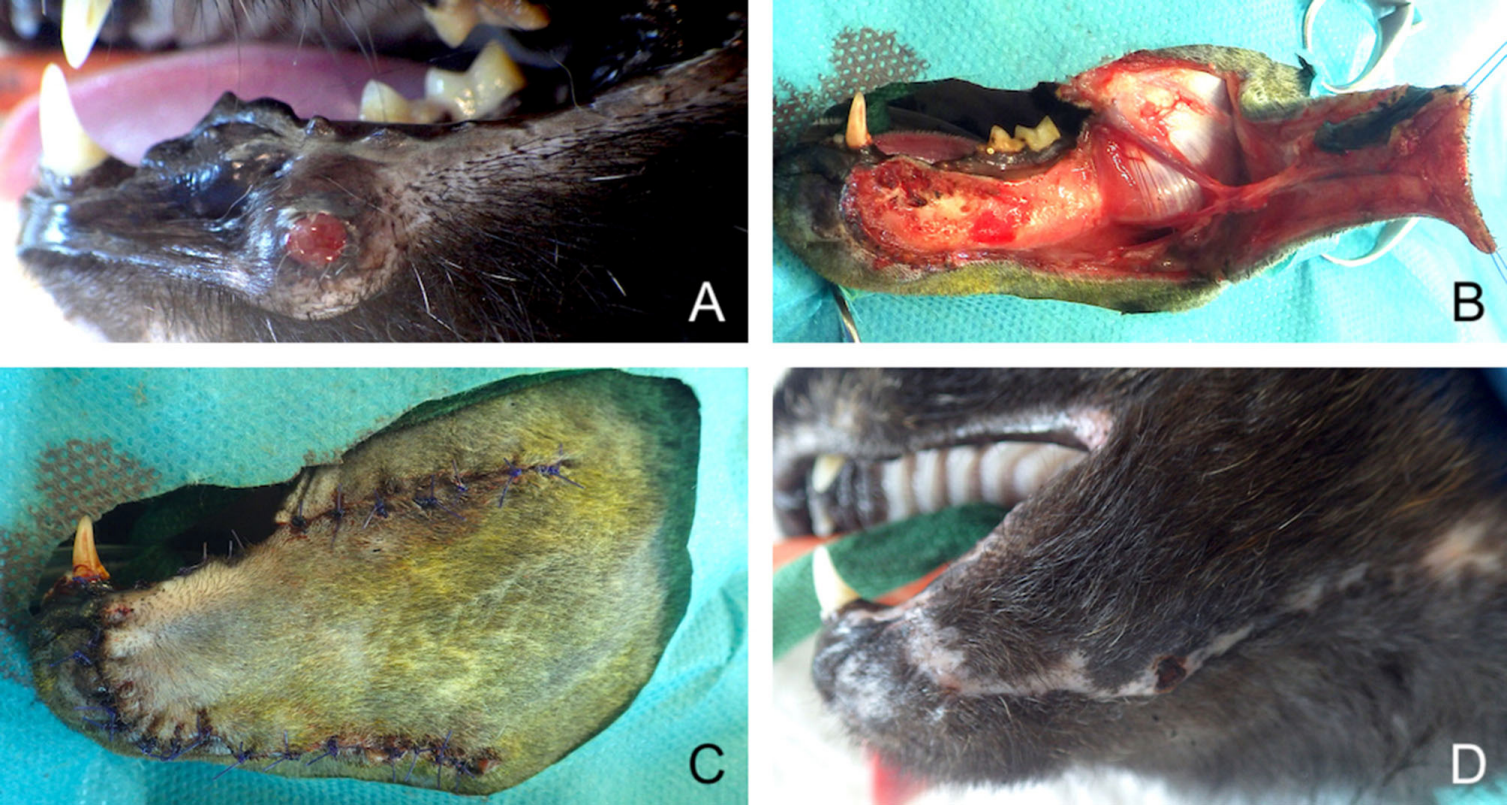
Full-thickness local advancement flaps for closure of a defect secondary to surgical removal of a recurring lower lip trabecular adenocarcinoma. **(A)** Preoperative aspect of the tumour. **(B)** Harvesting of the flap based on facial vascularisation. **(C)** Immediate postoperative aspect. **(D)** 1-month postoperative aspect.

***Full-thickness labial/buccal advancement flaps*** can be used to close a defect of the superior or the inferior lip. A skin incision is made parallel to the long axis of the defect either dorsally (maxillary defect) or ventrally (mandibular defect) and extended caudally as far as necessary to make use of tissue elasticity to stretch the flap over the defect. By doing so, the commissure of the lips is moved rostrally (Figure 6). For defects located on the rostral part of the lip, the base of the flap may not extend beyond the commissure provided enough tissue is available for tension-free closure. Only one longitudinal full-thickness incision of the lip is required, either dorsally for the superior lip or ventrally for the inferior lip. Opposite to the skin incision, the oral mucosa is incised away from the mucogingival junction to facilitate mucosal closure. For defects involving the middle or caudal part of the lip, the base of the flap needs to extend to the cheek or further caudally in the masseter region. Two slightly divergent full-thickness incisions are performed. One is ventrally or dorsally located and the second is at the level of the commissure. These full-thickness flaps are based either on the superior or inferior labial vascularization. The infraorbital/lateral nasal vessels (superior lip) or the caudal mental branch of the inferior alveolar artery (inferior lip) may need to be ligated and cut for flap mobilisation. As previously described, numerous anastomoses occur between these branches and the superior or inferior labial arteries forming a dense arterial network (70). When making the full-thickness incision at the commissure of the lip toward the cheek, care is taken to preserve the angularis oris blood vessels. Further backwards, the parotid (Stensen) duct and the buccal ventral and dorsal branches of the facial nerve, running on the surface of the masseter, must be identified and preserved (Figure 7).

**Figure 8:**
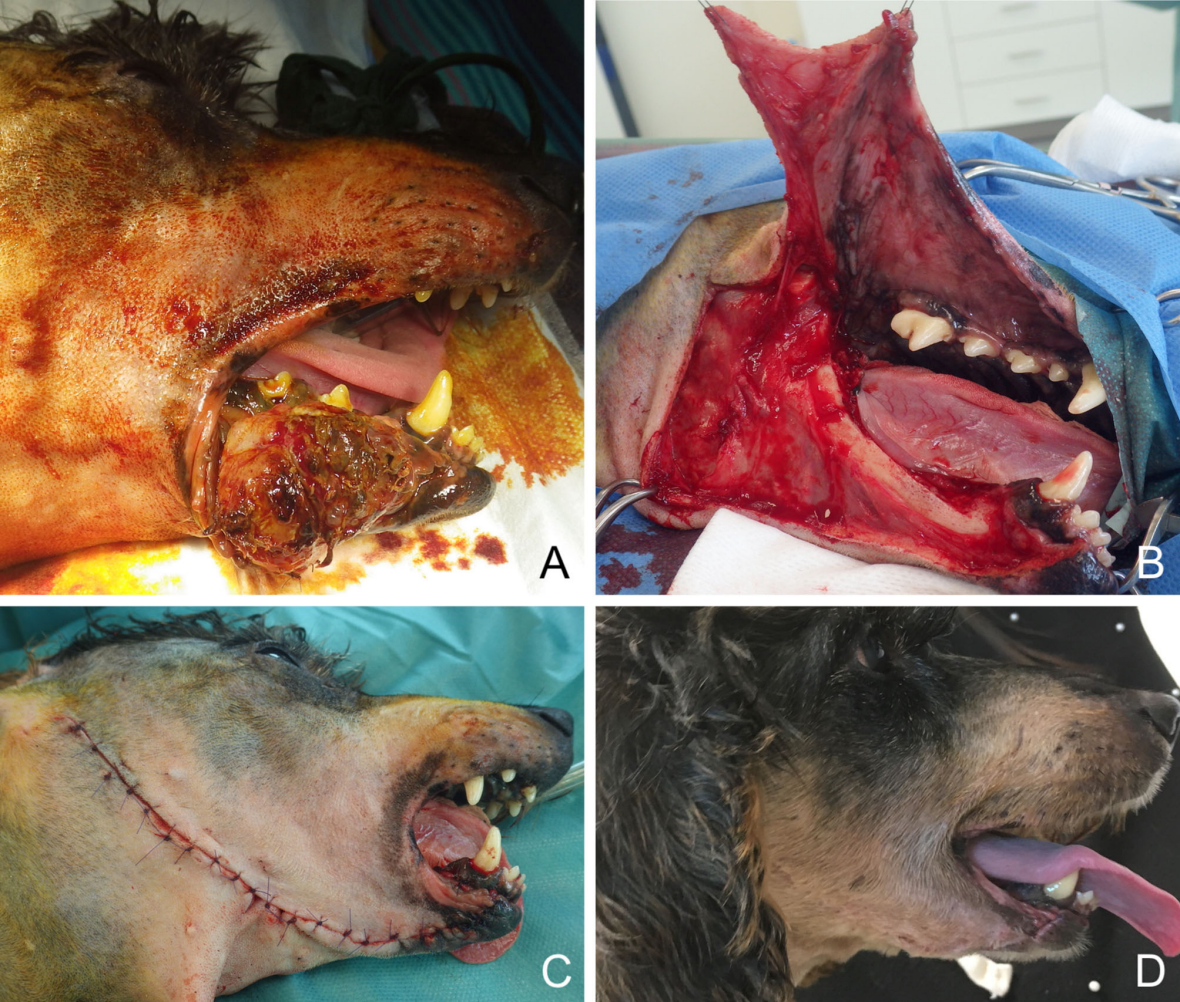
Full-thickness local rotation flap for closure of a lower lip defect in a dog. **(A)** Oral malignant melanoma of the lower rostral lip. **(B)** Harvesting of the flap. **(C)** Immediate postoperative appearance. **(D)** 6-months postoperative appearance.

A ***full-thickness buccal rotation flap*** is a semi-circular flap that rotates into the adjacent recipient bed. Although the flap is rotational in its direction, it also spans the defect by stretching the elastic tissues (76). The ideal defect for a rotation flap is triangular in shape. The flap is rotated at a maximum of 90° from the axis of the defect. The height-width ratio of the triangle ideally should be 2:1. The length of the flap should be 4 times the width of the base of the triangular defect (76). Large lip defects with insufficient remaining tissue on the same jaw can be reconstructed with a rotation flap using the lip of the opposite jaw. This technique has been described both for the superior and inferior lips as well as for nasal reconstruction (41, 42, 95, 101). Through the rotation of tissue, the commissure is moved forward dorsally or ventrally resulting in a slight facial asymmetry with a unilateral shortening of the opening between the lips (*rima oris*). This is considered a minor cosmetic change (Figure 8).

**Figure 9:**
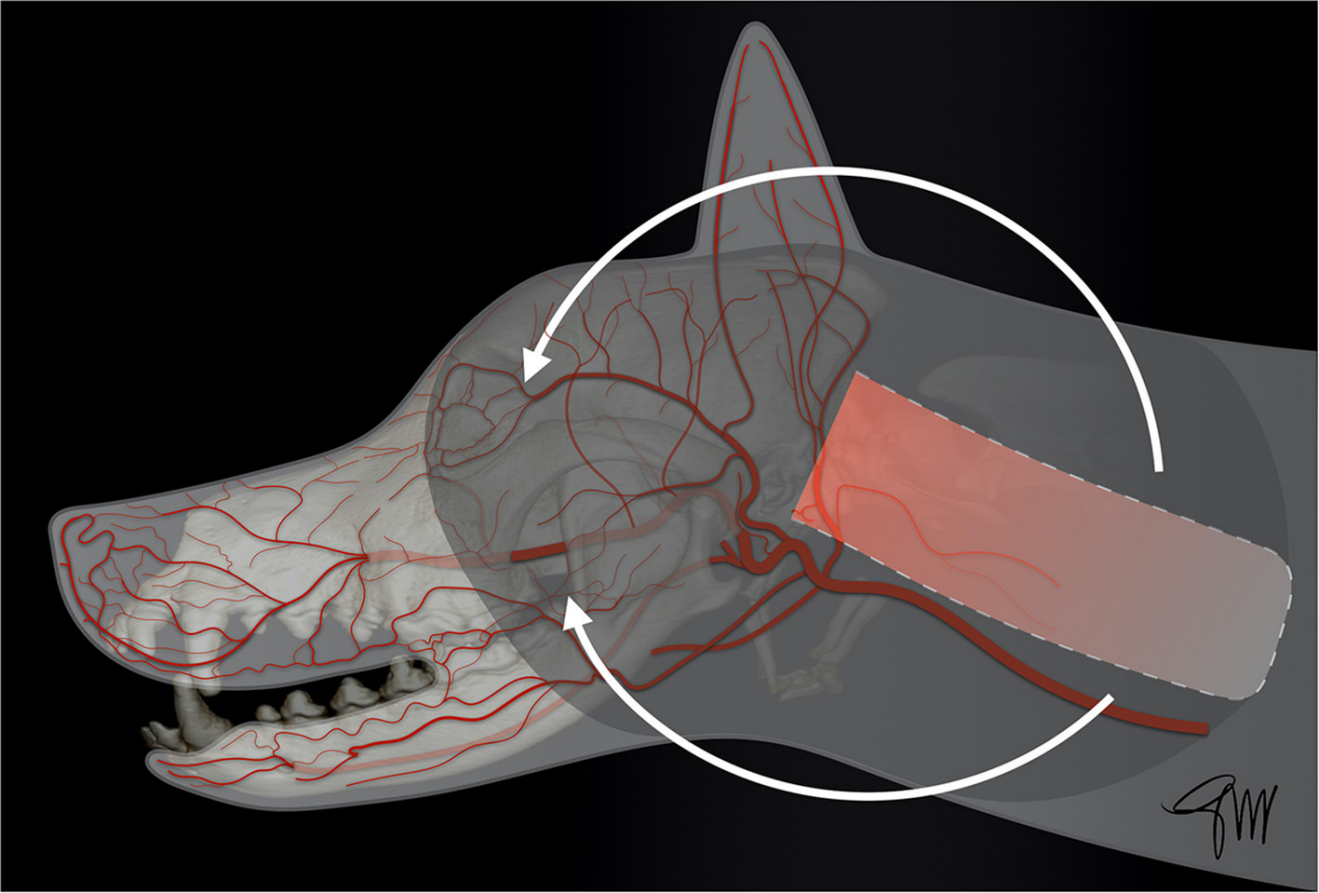
Drawing depicting the surgical limits and extent of rotation of a caudal auricular myocutaneous axial-pattern flap.

The ***caudal auricular myocutaneous axial-pattern flap*** can be used to close defects in the caudal part of the face, namely the cheek, the orbital area, and the parietotemporal area (upper face) (102). The base (cranial border) of the flap is centered between the lateral aspect of the wing of the atlas and the vertical ear canal. It is based on the sternocleidomastoideus branches of the caudal auricular artery and vein, providing blood supply to the platysma muscle, with perforating branches into the subcutaneous tissue, and the overlying skin. The plane of dissection lies just over the cervical neck fascia (Figure 9). Anastomoses with the superficial cervical artery may extend the flap design up to the spine of the scapula (67, 103). Similar anatomy has been described in dogs and cats (68). In the original experimental study and description of the technique in dogs a mean flap length survival of 85.2% was reported (103). In a similar study in cats using 3 cm-wide flaps and various lengths of 6, 9, and 12 cm, distal flap necrosis was reported in 75% of the cases, with a mean survival length of 93.8, 81.9, and 84.4%, respectively. Following this experimental study, two client-owned cats experienced a good outcome after transposition on aural and periorbital defects, but using wider flap dimensions of 4 × 10, and 5 × 12 cm, respectively (67). Subsequently, the caudal auricular myocutaneous axial-pattern flap has also been successfully used to close defects following exenteration, or upper eyelid reconstruction in dogs, and cats (104–107). Recently, a multicentric retrospective study in 16 dogs and 12 cats has shown a relatively high complication rate with necrosis of the distal aspect of the flap in 62.5% of the dogs and 41.7% of the cats, requiring revision surgery in 72.7% of the dogs and 50% of the cats with complications. Flap necrosis involved 10–25% of the flap in dogs and 25–50% of the flap in cats. It should be noticed that flap dimensions in this retrospective study was superior to that of the experimental study, with a mean width of 6.5 cm in dogs and 5 cm in cats and a mean length of 19 cm in dogs and 16 cm in cats. Decreasing the length of the outlined flap, as well as avoiding as much as possible any factors that could impair blood supply (e.g., excessive tension, or torsion of the pedicle, thrombosis, infection) is recommended in order to enhance the flap reliability (102).

**Figure 10:**
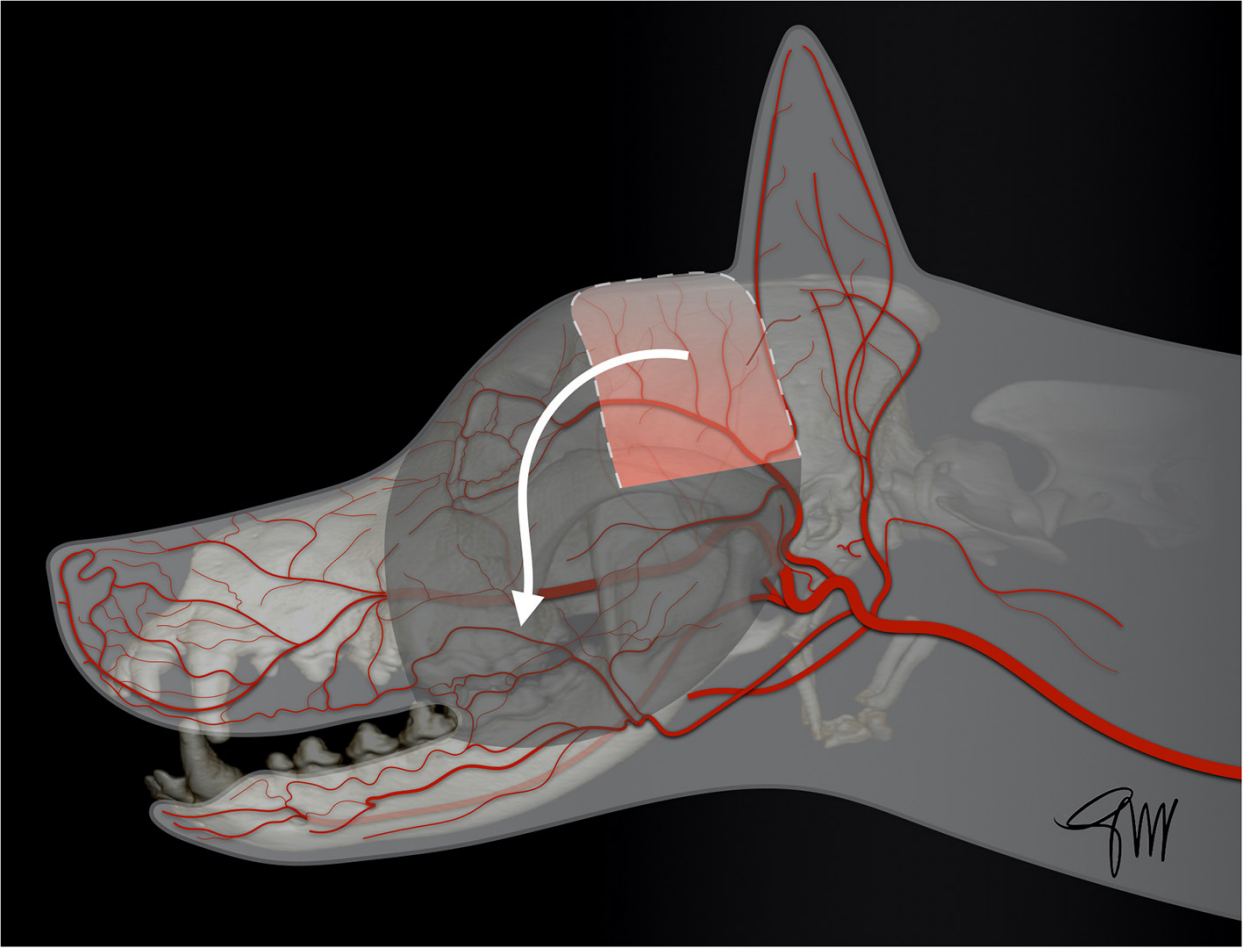
Drawing depicting the surgical limits and extent of rotation of a superficial temporal myocutaneous axial-pattern flap.

**Figure 11:**
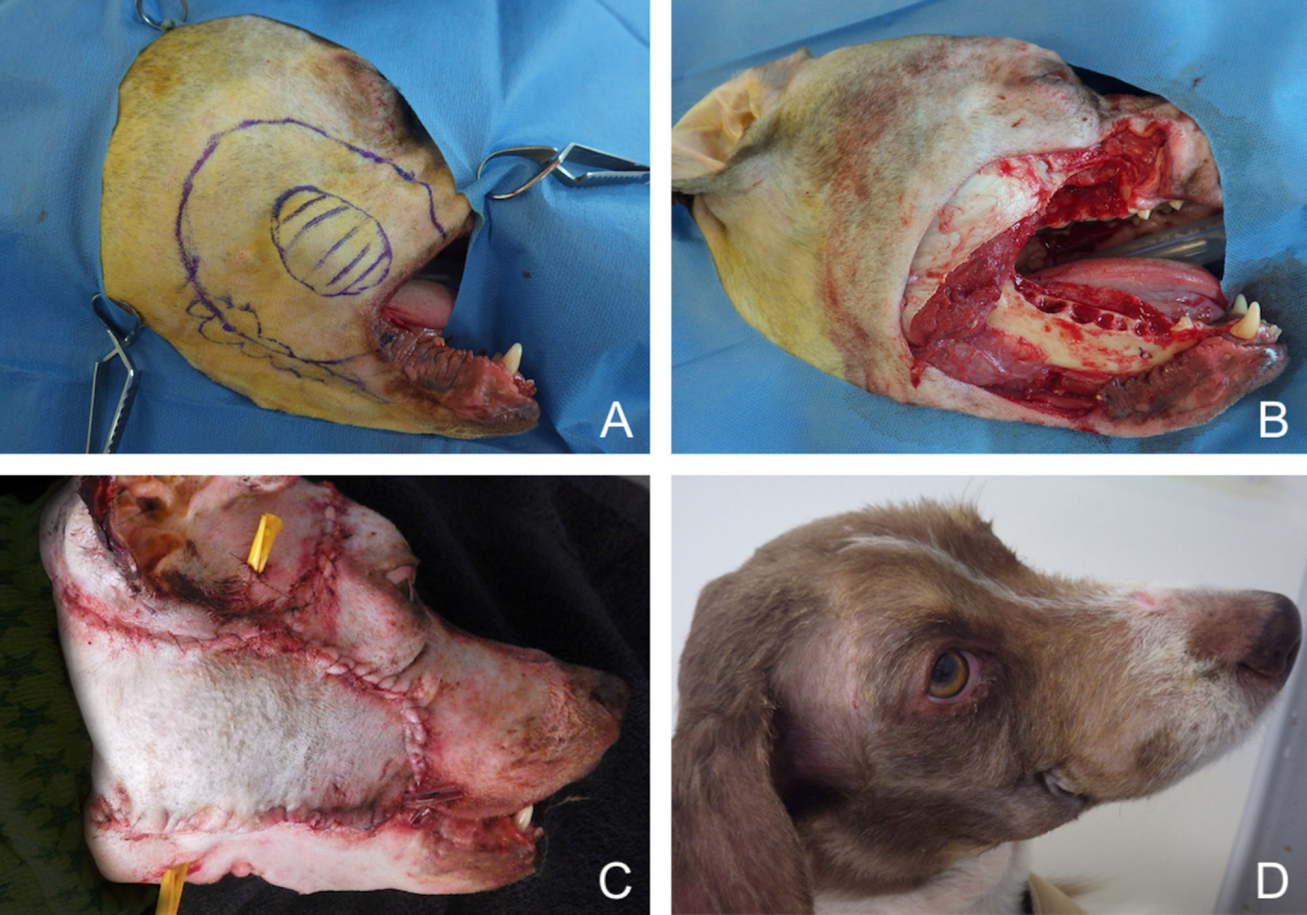
Closure of a large facial composite defect with a superficial temporal myocutaneous axial-pattern flap in a dog with an invasive oral malignant melanoma. **(A)** Surgical planning. **(B)** Peri-operative aspect after removal of the cheek, caudal maxilla. **(C)** Immediate postoperative aspect. **(D)** 1.5-months postoperative aspect.

The ***superficial temporal myocutaneous axial-pattern flap***, first described by Fahie et al. (68) in dogs and cats, is based on the cutaneous branch of the superficial temporal artery, originating at the base of the zygomatic arch (68). The anatomical landmarks and viability of the flap have been experimentally studied in dogs (69). The base of the flap corresponded approximately to the length of the zygomatic arch, as the width of the flap is limited rostrally by the eye and caudally by the ear (Figure 10). At 7 days post-operatively, flaps extending to the midline of the head showed significantly better length survival (91.8%) than flaps extending to the contralateral zygomatic arch (69.5%) or flaps in the control group with vessel ligation. No palpebral deficit was reported though a cranial branch arising from the auricular plexus was transected to facilitate flap rotation. The donor site was closed by a simple cranial advancement (myo)cutaneous flap. Due to the risk of extensive necrosis of the distal tip, it was concluded that extending the flap beyond the contralateral mid-dorsal orbital rim, and rotation >90° was not recommended (69). Subsequently, the superficial temporal myocutaneous flap was successfully used in client-owned dogs and cats to close defects affecting the maxillary, orbital or nasal area (69). The rostral extent of the flap in the maxillary area is dependant of the specific head shape. Dogs and cats with a short nose and a large rounded head may benefit the most from this technique (Figure 11).

**Figure 12:**
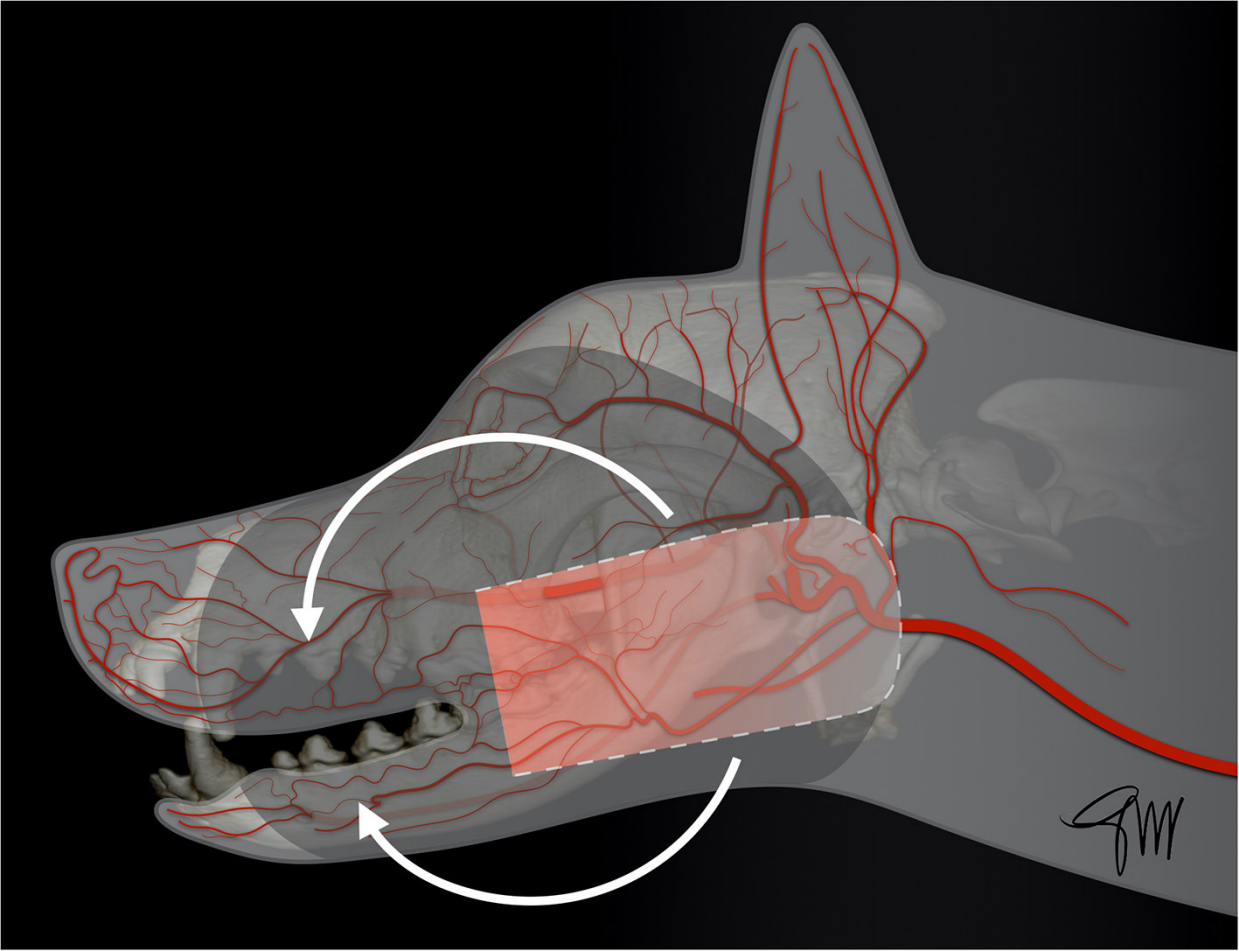
Drawing depicting the surgical limits and extent of rotation of a facial (angularis oris) transposition flap.

**Figure 13:**
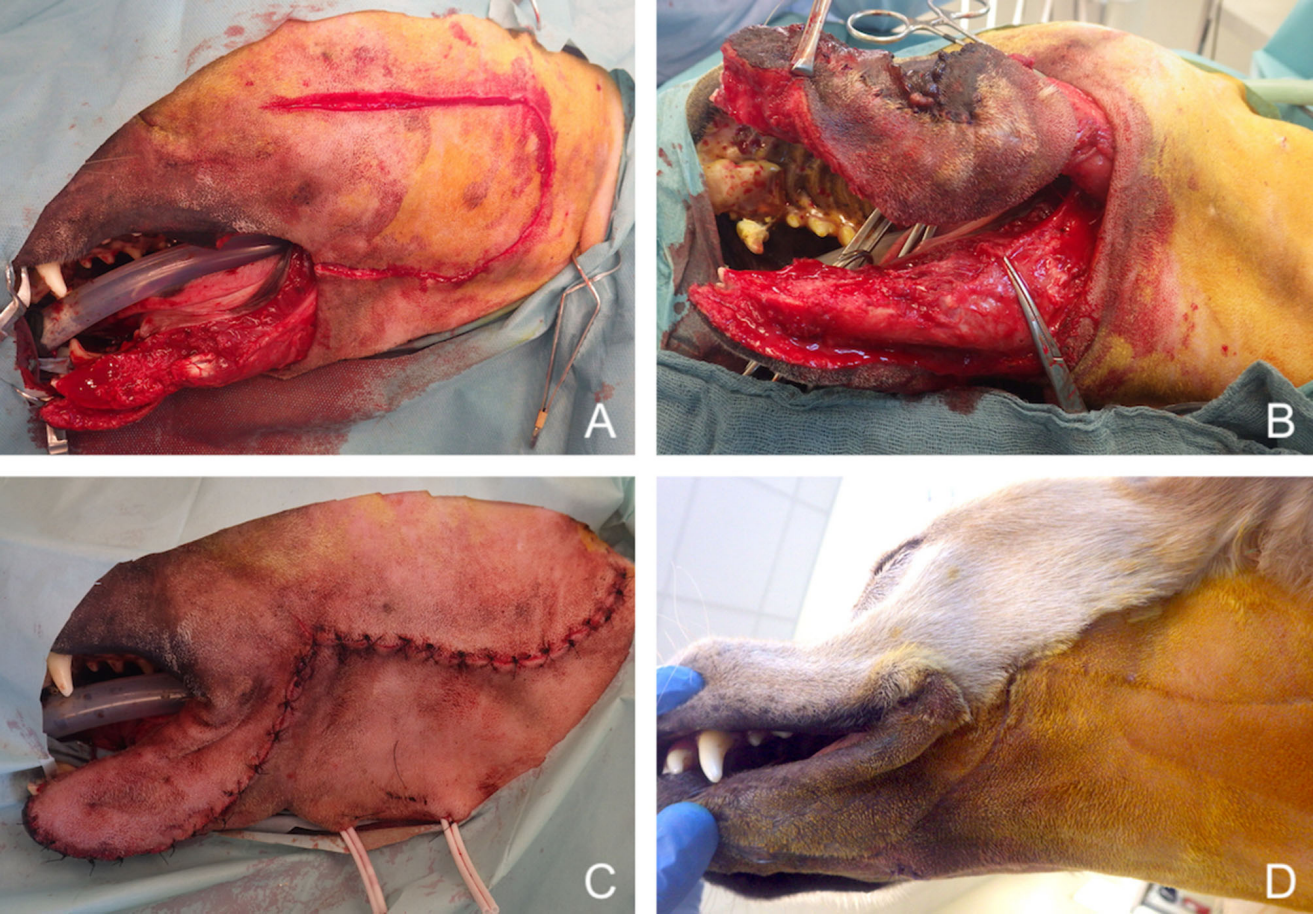
Reconstruction of a composite defect of the lower face with a facial (angularis oris) myocutaneous flap. **(A)** Composite resection of the body of the mandible and lower lip in a dog presenting with a neurofibrosarcoma. **(B)** Surgical preparation of the flap. **(C)** Immediate postoperative appearance. **(D)** 9-months postoperative appearance.

**Figure 14:**
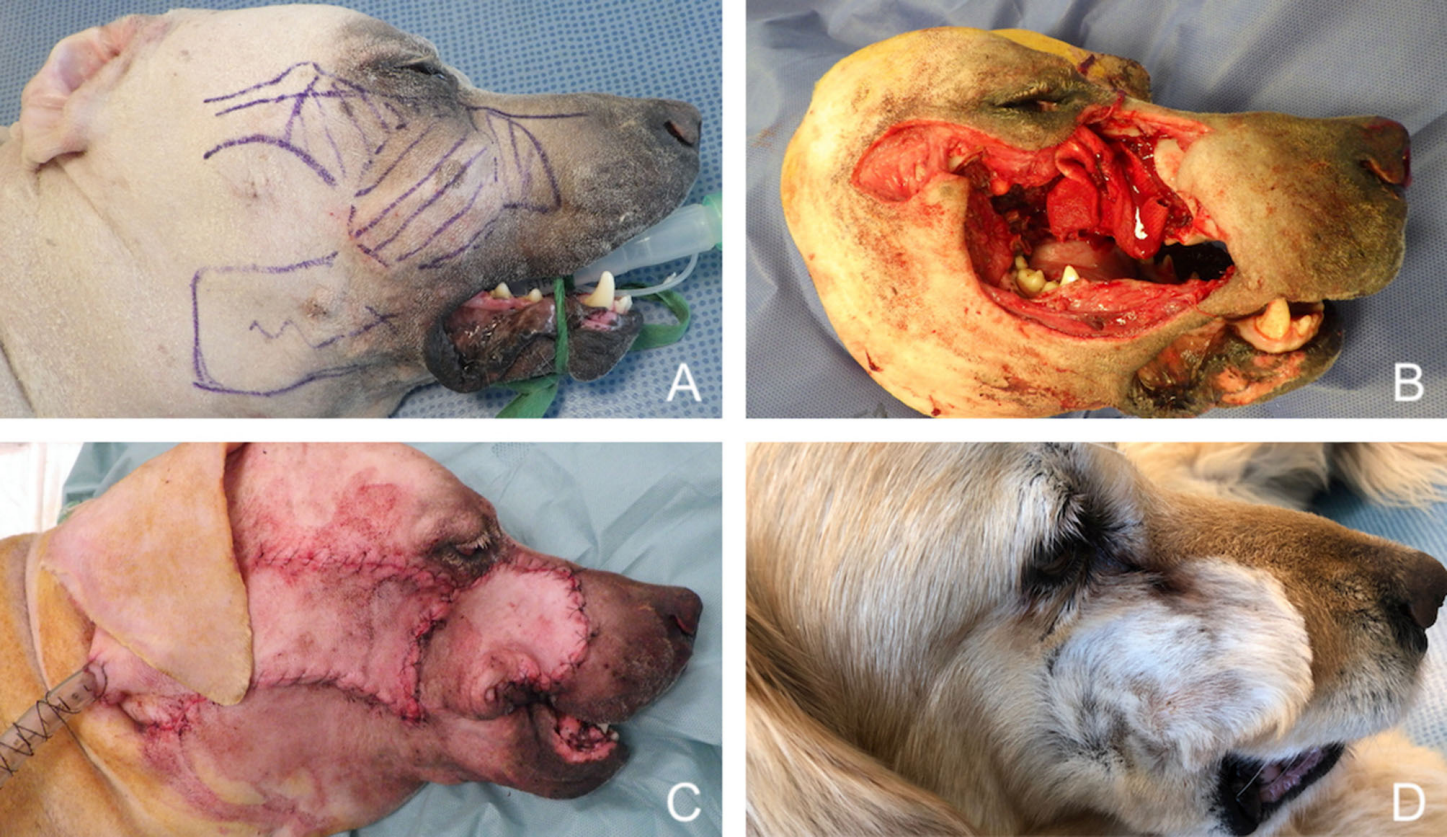
Reconstruction of a composite defect of the mid face with a facial (angularis oris) myocutaneous flap. **(A)** Surgical planning of the surgery delineating the surgical limits (oblique dash line) and the outline of the flap. **(B)** Composite resection of the orbitozygomaticomaxillary area in a dog presenting with an osteosarcoma. **(C)** Immediate postoperative appearance. **(D)** 4-months postoperative appearance.

The ***facial/angularis oris myocutaneous transposition flap*** is a very versatile flap enabling the closure of facial soft tissue defects in dogs and cats (108–112). It can be used to close both maxillary and mandibular defects. The anatomical boundaries of this U-shaped skin flap are the lip commissure at the level of the medial canthus of the eye (base), the ventral aspect of the zygomatic arch (dorsally), the ventral margin of the caudal mandible (ventrally) and the wing of the atlas (caudally) (111). The base of the flap is vascularized by direct cutaneous arteries arising from the superior, inferior labial arteries, and the angularis oris artery, which are branches of the facial artery; these branches penetrate into and through the platysma muscle (111). The distal end of the flap is also vascularized by two direct arteries entering in a rostral direction: the transverse facial artery and a cutaneous branch of the masseteric artery; they anastomose with a separate cutaneous branch of the angularis oris artery running caudally toward the ear canal area (111, 112) (Figure 12). Flap design must be carefully planned prior to the resective surgery by measuring the resulting defect size and its distance from the base of the flap to ensure full coverage of the defect. The boundaries of the resection and of the flap are delineated with a sterile surgical marker. A template created by cutting a piece of surgical drape may be used to help design the flap. Skin and superficial muscle are incised with a scalpel blade and dissection is performed deep under the platysma using blunt dissection scissors in caudal to rostral direction. Care is advised when approaching the angle of the mouth to preserve angularis oris vessels. A dorsoventral line drawn from the medial canthus and perpendicular to the axis of the mandibular body can be used as a safe rostral limit (111). Two small series of angularis oris myocutaneous transposition flap have been published in dogs (108, 112). Most of the cases were used to treat middle-face defects with a flap rotated dorsally. Losinsky et al. (112) reported the outcome of 9 flaps in 8 dogs. Results were good with no major complications observed. Partial incisional dehiscence of the distal end of the flap was noticed in 3 dogs with 2 of the dogs showing small area of necrosis (<1 cm); the defect healed by second intention in one of the dogs or after minor revision surgery in two of them (112). Frasson et al. (108) reported the outcome of the flap in 6 dogs. Three of the 6 flaps showed minimal suture dehiscence but all flaps healed without necrosis of any portion of the flaps and did not require revision surgery (108). In both series, the caudal limit of the flap was either the vertical external ear canal or the wing of the atlas depending of the defect to be covered (Figures 13, 14). Distal flap dehiscence and/or necrosis was not associated with a more caudal extent of the flap. Mild to moderate flap oedema was the most common minor complication (108, 112). A modification of this flap has recently been documented in a small series of three dogs to reconstruct large palatal defect. A full-thickness bridging incision is made ventrally at the level of the last mandibular molar tooth to allow flap transposition into the oral cavity. Though hair regrew on the surface of the flap within the oral cavity, no major complication was reported in this short series (113).

The ***temporal myofascial flap*** was described in the dog by Tomlinson and Presnell (114) for closure of a 3-cm-diameter orbitonasal defect secondary to exenteration of the orbit and radiation therapy (114). Another paper has reported the use of this flap for closure of a 15 mm-diameter caudal oronasal fistula in a canine patient after caudal maxillectomy with exenteration and radiation therapy (115). Additionally, an experimental study published in 1996 assessed the feasibility and the outcome of this technique following total unilateral maxillectomy in thirty cats (116). The myofascial flap is based on the rostral and caudal deep temporal arteries. A curvilinear skin incision is made between the orbit and the ear. The temporal fascia covering the temporal muscle is exposed. The temporalis is harvested circumferentially from the temporal fossa by incising its attachment to the temporal line up to the sagittal crest dorsally, to the nuchal crest caudally, to the zygomatic arch laterally and to the caudal orbital margins rostrally (114–116). During elevation of the flap, the deep temporal artery and vein located ventro-medially, as well as the attachments of the muscle over the coronoid process of the mandible are carefully preserved. Transposition of the flap in the oral cavity is performed after tunnelization under the orbit. The flap is sutured over the defect with the fascia facing the oral cavity (116). Ostectomy of a portion of the zygomatic arch, or osteotomy of the coronoid process of the mandible have been suggested to facilitate mobilization of the flap (114, 116). Good clinical outcome has been reported in cats despite a longer healing time compared to humans. Coverage by a smooth oral mucosa was achieved in experimental cats 18–24 weeks after surgery (116). Indications of temporalis muscle flap in humans comprise the repair of defects involving the ipsilateral maxilla and palate, the lateral oropharyngeal wall and the retromolar area (117, 118). So far, there is limited documentation of the clinical use of this flap to repair large oronasal fistula in dogs and cats.

More distant axial-pattern flaps such as the ***superficial cervical myo-cutaneous flap*** have been suggested, though not yet fully investigated for facial reconstructions to the authors' knowledge (79). As transposition to the caudal oral cavity has already been described (see previous section), the reconstruction of defects involving the upper face and caudal aspect of the lower and midface (cheek) might be possible (94).

## Decisional Algorithm

**Figure 15:**
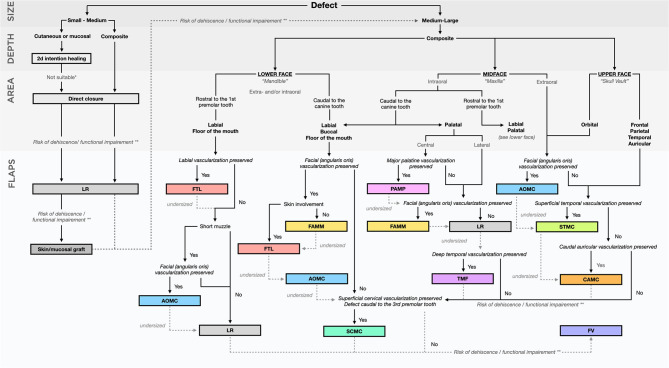
Decisional algorithm. *e.g., risk of periorbicular fibrosis/retraction, infection, bone exposure. **e.g., risk of oronasal communication, occlusal trauma, inability to eat, wound dehiscence, upper airways stenosis/obstruction.

To help the clinician planning the reconstructive phase following resective oromaxillofacial surgery, we proposed a decisional algorithm based on the current literature (Figure 15). When considering the reconstruction of oromaxillofacial defects, the easiest and most suitable technique by increasing order of difficulty should be considered first (53, 60). As the complexity of procedure may further impact the duration of the anaesthesia and the outcome, the clinician should also focus on its reliability as well as its time- and cost-effectiveness. The decisional process starts with a good appraisal of the resective procedure in order to plan the reconstructive modality accordingly (13–29). Small defects involving superficial soft tissue layers can sometimes be managed by second intention healing, providing inspection and special care of the wound are satisfactory, with no high risk of fibrosis or site infection. Direct closure, local random flaps or skin/mucosal grafts are generally preferred. Some deeper, moderately extensive defects may also be advantageously treated by direct closure or local random flaps whenever possible. For more severe (medium to large) defects, locoregional axial pattern flaps constitute the technique of choice. They may be combined with other techniques. They enable the restoration of an anatomic barrier between the oral cavity, the nasal cavity and/or the cutaneous layer in order to preserve function. The choice of the proper flap is dependent on the location of the defect. As a general rule, the closest available flap is preferred as a more distant harvesting site may require a longer flap to reach the defect to be covered and may be associated with a higher risk of distal flap necrosis. When a specific procedure may lead to an excessive risk of dehiscence (e.g. undersized, compromised vascularization, excessive tension), or when functional impairment may be associated, the next flap in the decisional algorithm should be considered. Flaps with low scientific support, and those necessitating highly trained teams and fully-equipped facilities (e.g., free vascularized flaps) are considered at last resort. Flaps based on the facial artery seem the more versatile and reliable and, due to their anatomical proximity, are currently used in most of the situations in first-intent closure of oromaxillofacial defects.

## Perioperative Care

Perioperative care is considered of fundamental importance in head and neck surgery and has been shown to influence the outcome in humans (119, 120). As little is documented in veterinary medicine, the veterinary patient may benefit from human recommendations.

### Antibiotics

Antibiotic prophylaxis is recommended for orofacial reconstructive surgery with exposure to the oral or nasal environment (clean-contaminated surgery) because of the high risk of surgical site infection (119, 121, 122). Type and duration of antibiotic treatment has been debated in humans. Currently, ampicillin-sulbactam, amoxicillin-clavulanate and cefazolin are considered antibiotics of choice and are used postoperatively up to 24–48 h (75, 119–123). Some studies have shown that clindamycin should be avoided postoperatively in free flap surgery in humans as it was found associated with a poorer outcome due to its ineffectiveness against gram negative bacteria (121, 122). Reports on the use of antibiotics in head and neck surgery in dogs and cats are scarce. Human recommendations on the use of perioperative antibiotics are followed in some reports (6, 88, 112, 113). In a large retrospective study concerning axial pattern flaps in dogs and cats antibiotics were used postoperatively in 88% of the animals and surgical site infections was reported in 28% of the patients (81). Preoperative and postoperative antibiotic treatments were not found to have a significant impact on the outcome nor on the complication rate in this retrospective study (81). As no specific study has been conducted in veterinary medicine, our recommendation is to follow human protocols on antibiotic use when performing head and neck reconstructive surgery.

### Drain Placement

Head and neck operations are associated with clinically significant postoperative morbidities such as haematoma, seroma, wound infection, and flap complications. Surgeons may choose to use *drainage systems* to reduce these complications (124, 125). However, the decision to place a drain is mostly based on individual cases and surgeons' experience and preference (126). A recent national survey in Ireland has shown that there were poorly defined guidelines and considerable heterogeneity amongst surgeons in term of indication for insertion (127). No significant difference in complication rate was found between active compared to passive drainage after free flap reconstruction in head and neck surgery in humans (124). Great heterogeneity amongst studies is also found in the veterinary literature. In a recent retrospective study on subdermal skin flaps in dogs and cats, an active drainage was placed in 11% of the cases and a passive one in 26% of the cases according to the surgeon preference (78). In a retrospective study on axial pattern flap in dogs and cats, wound drains were used in 66% of the dogs and 38% of the cats and removed after a median of 2 days in dogs and 3 days in cats (81). The placement of a drain and the duration of the drain were not shown to have a significant impact on the outcome nor the complication rate (81). In another study on caudal auricular axial pattern flap in 16 dogs and 12 cats, an active drain was used only in 18% of the dogs and 8% of the cats (102). Therefore, until studies will have been performed to compare the outcome with or without drains as well as the influence of the type of drain, the decision will remain in the surgeon's hands based on the individual case.

### Pain Management

Efficient pain management relies on a multimodal approach combining strong opioids, non-opioid analgesics, and peripheral or neuraxial local anaesthetics acting on different sites of the pain pathway (82, 89). In our institution, and in agreement with others, perioperative analgesia is achieved through a combination of locoregional anaesthesia using ropivacaine and constant rate infusion of drugs (opioids, lidocaine, ketamine, or dexmedetomidine) for the first 24–48 h (6, 62, 78). Other drugs that may be used include non-steroidal anti-inflammatory drugs and gabapentinoids. In a recent systematic review in humans, gabapentin, and pregabalin showed significant beneficial effect on perioperative pain relief and analgesic consumption in head and neck surgery procedures within the first 24 h (90, 91). The degree and duration of the postoperative pain management is based on the type of surgery and on the patient's pain score according to current recommendations (92). The less painful the animal, the faster food intake, and recovery are.

### Nutrition

Patients suffering from oral tumour may have already lost weight at the time of surgery. Preoperative malnutrition in humans is a well-documented risk factor for perioperative complications and poor outcome (81). Decision to resume oral intake varies between surgeons and institutions but is commonly 5–7 days postoperatively in surgery involving the upper aerodigestive tract in humans (119). As placement of an esophageal feeding tube at time of surgery is a quick and straightforward procedure, we routinely use them in maxillofacial surgery. Large retrospective studies in dogs and cats have shown that most complications are minor (tube displacement, infection of stoma) and can be easily managed (128, 129). A feeding tube allows fast recovery and food and drug administration to the animal while preserving the oral cavity from any mechanical activity. Though there is no study to evaluate postoperative outcome with or without placement of a feeding tube, in agreement with other authors we favour this approach (6, 88, 89).

## Conclusion

Oromaxillofacial surgery has become a specific branch of veterinary surgery, which must take into account specific oral anatomic and functional considerations. Management of large defects resulting from oncologic resective procedures requires excellent knowledge of the head and neck anatomy, and of the different reconstructive options available. Locoregional axial-pattern flaps constitute an important component of the therapeutic armamentarium in oromaxillofacial surgery as they allow for immediate reconstruction of the defects while preserving function and achieving good cosmetic results. These are key elements for the owner's acceptance of the resective surgery. Recently, emphasis has been placed on locoregional mucosal or skin axial pattern flaps based on branches of the facial artery (angularis oris, superior labial artery). These flaps seem versatile and reliable and, due to their anatomical proximity, are best used for reconstruction of orofacial defects. More retrospective studies are needed to fully evaluate the full potential of these techniques. Most of the orofacial reconstructive techniques used are aimed at closing the defect and focus on soft tissue reconstruction. The development of reliable and cost-effective flaps, allowing one-step composite reconstruction, appears to be the next step for the future of maxillofacial reconstructive surgery in small animals.

## Author Contributions

MG: literature searches, drafting and critical revision for intellectual content of the manuscript, idea and production of the schemes and diagram. DR: review of the article. PH: idea to write a review on locoregional flap reconstruction, proposition to submit to Frontiers in Veterinary Science, literature searches, drafting, critical revision for intellectual content, editing of the manuscript, and selection of the figures.

## Conflict of Interest

The authors declare that the research was conducted in the absence of any commercial or financial relationships that could be construed as a potential conflict of interest.

## Abbreviations

AOMC: Angularis Oris MyoCutaneous
CAMC: Caudal Auricular MyoCutaneous
FAMM: Facial Artery MyoMucosal
FTL: Full-Thickness Local
FV: Free (vascularized)
LR: Local Random
PAMP: Palatine Artery MucoPeriosteal
SCMC: Superficial Cervical MyoCutaneous
STMC: Superficial Temporalis MyoCutaneous
TMF: Temporal MyoFascial.
